# APOE E4 is associated with hyperlipidemia and obesity in elderly schizophrenic patients

**DOI:** 10.1038/s41598-021-94381-4

**Published:** 2021-07-20

**Authors:** Wei Li, Fengju Liu, Rui Liu, Xinmei Zhou, Guanjun Li, Shifu Xiao

**Affiliations:** 1 Department of Geriatric Psychiatry, Shanghai Mental Health Center, Shanghai Jiao Tong University School of Medicine, Shanghai, 200030 China; 2 Alzheimer’s Disease and Related Disorders Center, Shanghai Jiao Tong University, Shanghai, China; 3 Department of Early Psychotic Disorder, Shanghai Mental Health Center, Shanghai Jiao Tong University School of Medicine, Shanghai, China; 4 Department of General Practice, Zhongshan Hospital, Fudan University, Shanghai, China

**Keywords:** Genotype, Risk factors, Signs and symptoms

## Abstract

Obesity is a critical issue in patients with schizophrenia, which is considered to be brought about by both environmental and genetic factors. Apolipoprotein E (APOE) gene polymorphisms might be involved in the pathogenesis of schizophrenia, however, the effect of APOE gene polymorphism on obesity has never been investigated in Chinese aging with schizophrenia. This cross-sectional study was to investigate the effect of obesity on cognitive and psychiatric symptoms in elderly participants with schizophrenia. At the same time, we also discussed the inner link between APOE E4 and obesity. 301 elderly participants with schizophrenia and 156 normal controls were included in the study. Their cognitive function was assessed using the Montreal Cognitive Assessment (MoCA), psychiatric symptoms were assessed using the Positive and Negative Syndrome Scale (PANSS), and APOE gene polymorphism was determined by polymerase chain reaction (PCR). The prevalence of obesity in elderly schizophrenic patients and healthy controls accounted for 15.9% (48/301) and 10.3% (16/156), respectively, with no statistically significant difference. By using stepwise linear regression analysis, we found that elevated fasting blood glucose, hypertension, and hyperlipidemia were risk factors for obesity in elderly schizophrenic patients. Although there was no direct correlation between APOE E4 and obesity in patients with schizophrenia, it was significantly correlated with hyperlipemia(r = − 0.154, p = 0.008), suggesting that APOE E4 may induce obesity in elderly patients with schizophrenia through hyperlipemia, However, the above conclusions do not apply to the normal elderly. What’s more, we did not find a link between obesity and cognitive function or mental symptoms for both patients with schizophrenia and normal controls. APOE E4 is associated with hyperlipidemia in elderly schizophrenic patients, which may be a risk factor for obesity, however, the above conclusion does not apply to the normal elderly.

## Introduction

Obesity is a critical issue in patients with schizophrenia, which can adversely affect the risk of cardiovascular disorders, adult‐onset diabetes mellitus, quality of life, non‐adherence with pharmacological interventions as well as psychiatric readmissions^1^. According to previous studies, obesity will affect 40–60% of people with schizophrenia^2^. Although some studies have shown that second-generation antipsychotics are the main cause of obesity^3–5^, others have shown that unhealthy lifestyle, socioeconomic disadvantages, and premorbid genetic vulnerabilities may also play an important role^6^.

Apolipoprotein E (APOE) E4 allele is the largest genetic risk factor for Alzheimer’s disease (AD)^7^, and it has been confirmed to be associated with the accelerated development of cognitive deficits and increased in myelin breakdown^8^. Due to a similar decline in specific cognitive domains, Harrington et al.^9^ first hypothesized that APOE might also play an important role in schizophrenia. However, the association between schizophrenia and APOE is complex, and the relevant conclusions are inconsistent, for example, a meta-analysis of 17 studies have shown that APOE E4 is a risk factor for schizophrenia^10^, while another meta-analysis of 28 association studies did not present the above conclusions^11^.

In the last decades, the APOE gene has been confirmed to be associated with obesity^12^, and many studies have shown that APOE is associated with obesity symptoms in AD patients^13–15^. However, only two studies have examined the association between ApoE and obesity in schizophrenic patients, one of which shows that the APOE expression in the hippocampus of schizophrenic patients is significantly higher than that in the control group^16^, while the other showed that APOE gene could influence the prevalence of diabetes and possibly overweight in psychiatric patients^17^, and the mechanism may involve lipid metabolisms^13^.

So far, there is no study on the relationship between obesity and apoE gene polymorphism in Chinese elderly schizophrenic patients, therefore, we conducted this cross-sectional study to investigate the relationship between APOE E4 and lipid metabolism and obesity.

## Materials and methods

### Participants

This cross-sectional study included 301 elderly patients with schizophrenia and 156 normal controls. Details can be found in our previous study^18–20^. The inclusion criteria were as follows: (1) aged 60 or more; (2) without major medical abnormalities, including unstable, acute, or life-threatening medical illness, and central nervous system diseases; (3) was able to cooperate and complete relevant inspections. Through face-to-face interviews and medical records, we obtained general demographic data (such as age, education, gender, BMI, duration of disease), daily living habits (smoking, drinking, drinking tea, physical exercise, hobby), disease history (hypertension, diabetes, and hyperlipidemia) and currently prescribed medicines (clozapine, olanzapine, quetiapine, risperidone, aripiprazole) of the subjects.

This study was subject to approval by the Research Ethical Committee of the affiliated mental health center of Shanghai Jiaotong University School of Medicine. And written informed consent was obtained from all participants before the study. All research processes were conducted under the principles of the Declaration of Helsinki.

### Clinical psychiatric assessment

Schizophrenia was diagnosed by two senior psychiatrists according to the International Classification of Diseases 10 diagnostic standards, while normal controls needed to exclude dementia and mild cognitive impairment (MCI).

### Cognitive assessment and psychotic symptoms assessment

The Montreal Cognitive Assessment (MoCA)^21^ was used to evaluate the cognitive function of all the participants, while the Positive and Negative Syndrome Scale (PANSS)^22^ was utilized to assess the symptoms and severity of schizophrenia. What’s more, we also used the Geriatric Depression Scale (GDS)^23^ to exclude depression.

### Genotyping of APOE and biochemical detection of blood lipids

All the subjects stopped eating after 9 p.m. and their peripheral blood was collected between 7:00 a.m. and 9:00 a.m (the next morning). Anticoagulant tubes and clot activating gel-containing serum separator tubes were used to assay blood indexes, such as cholesterol, triglyceride, fasting plasma glucose (FDG), high-density lipoprotein (HDL), and low-density lipoprotein (LDL). Genomic DNA was extracted from blood cells (after high-speed centrifugation) by using a Blood Genomic DNA Extraction Kit (Qiagen NV, Venlo, the Netherlands), and multiplex amplification refractory mutation system polymerase chain reaction (PCR) was used to determine the APOE genotype. According to the method described earlier^24^, APOE E4 included ε2/ε4, ε3/ε4, and ε4/ε4, while Non-APOE E4 types included ε2/ε2, ε2/ε3 and ε3/ε3.

### Statistical analysis

Continuous variables were expressed as mean ± SD and categorical variables were expressed as frequencies (%). The single sample Kolmogorov–Smirnov test was used to test whether the data conform to the normal distribution. Independent sample t-test was used to compare the data of normal distribution between the obesity group and the non-obesity group, Mann–Whitney U test was used to compare the data of non-normal distribution, while the Chi-square test was used to categorical variables between the two groups. Stepwise linear regression analysis for screening risk factors of obesity and partial correlation analysis was utilized to explore the association between APOE E4 and blood lipids (gender was controlled). All statistical analyses were performed using SPSS 22.0 (IBM Corporation, Armonk, NY, USA), and two-tailed tests were utilized at a significance level of p < 0.05.

### Ethics approval and consent to participate

All the subjects signed an informed consent form at the start of the study, and ethical approval was obtained from the Shanghai Mental Health Center.

### Consent for publication

Not applicable.

## Results

Table 1 displays the characteristic of subjects with different weight statuses (BMI ≥ 28 was considered as obesity). The prevalence of obesity in elderly patients with schizophrenia was 15.9% (48/301), and obesity patients were more likely to be female, with higher BMI, fasting blood glucose, and a higher proportion of diabetes, hypertension, and hyperlipidemia (p < 0.05), while there were no significant difference (p > 0.05) in age, education, duration of disease, triglyceride, total cholesterol, high-density lipoprotein, low-density lipoprotein, APOE E4, smoker, drinker, tea drinker, physical exercise, hobby, clozapine, olanzapine, quetiapine, risperidone, aripiprazole and scores of MoCA, GDS, and PANSS between the obesity group and the non-obesity group. The results of stepwise regression analysis showed that fasting blood glucose (t = 4.025, p < 0.001, 95% CI 0.333–0.970), hyperlipidemia (t = 3.445, p = 0.001, 95% CI 0.686–2.515) and hypertension (t = 2.499, p = 0.013, 95% CI 0.249–2.097) were risk factors for obesity. Table 2 presents the results. Then by using the partial correlation analysis and controlling gender, we found that APOE E4 was associated was hyperlipidemia (r = − 0.154, p = 0.008), but not with hypertension or diabetes (p > 0.05).

**Table 1:** General demographic data of the Chinese elderly with schizophrenia based on obesity.

Variables	Obesity (N = 48)	Non-obesity (N = 253)	t or X^2^	p
Age, y	67.54 ± 7.520	67.25 ± 6.487	0.275	0.783
Education, y	7.25 ± 3.540	8.17 ± 3.725	− 1.577	0.116
Duration of disease, y	35.04 ± 12.976	36.39 ± 13.265	− 0.645	0.519
BMI, kg/m^2^	30.17 ± 2.500	22.62 ± 3.178	15.575	< 0.001*
Fasting blood glucose, mmol/L	6.20 ± 2.031	5.36 ± 1.233	3.846	< 0.001*
Triglyceride, mmol/L	1.48 ± 0.571	1.37 ± 0.866	0.905	0.366
Total cholesterol, mmol/L	4.68 ± 1.215	4.73 ± 0.987	− 0.298	0.766
High density lipoprotein, mmol/L	1.23 ± 0.401	1.31 ± 0.408	− 1.207	0.228
Low density lipoprotein, mmol/L	2.86 ± 0.989	2.74 ± 0.750	0.934	0.351
Male, n (%)	19 (39.6)	142 (56.1)	4.438	0.041*
APOE E4, n (%)	9 (18.8)	47 (18.6)	0.001	1.000
Hypertension, n (%)	26 (54.2)	85 (33.6)	7.334	0.009*
Diabetes, n (%)	24 (50.0)	54 (21.3)	17.257	< 0.001*
Hyperlipidemia, n (%)	26 (54.2)	94 (37.2)	4.871	0.036*
Smoker, n (%)	14 (29.2)	83 (32.8)	0.245	0.737
Drinker, n (%)	5 (10.4)	28 (11.1)	0.017	1.000
Tea drinker, n (%)	10 (21.3)	56 (22.1)	0.017	1.000
Physical exercise, n (%)	16(33.3)	79 (31.2)	0.083	0.866
Hobby, n (%)	15 (31.2)	94 (37.2)	0.609	0.513
Clozapine, n (%)	4 (8.3)	44 (17.4)	2.470	0.135
Olanzapine, n (%)	14 (29.2)	68 (26.9)	0.107	0.727
Quetiapine, n (%)	7 (14.6)	35 (13.8)	0.019	0.824
Risperidone, n (%)	13 (27.1)	74 (29.2)	0.092	0.863
Aripiprazole, n (%)	9 (18.8)	46 (18.2)	0.009	1.000
MoCA	13.60 ± 6.180	13.89 ± 7.047	− 0.249	0.803
GDS	10.23 ± 6.245	10.17 ± 5.820	0.055	0.957
PANSS total score	63.84 ± 19.737	64.64 ± 22.038	− 0.222	0.825
Positive score	11.60 ± 6.310	12.20 ± 5.997	− 0.586	0.558
Negative score	17.81 ± 7.664	18.92 ± 8.730	− 0.765	0.445
General score	33.42 ± 9.818	33.13 ± 11.039	0.158	0.874

*BMI* body mass index, *MoCA* Montreal Cognitive Assessment, *GDS* Geriatric Depression Scale, *PANSS* Positive and Negative Syndrome Scale.

*Means p < 0.05.

**Table 2:** The results of Stepwise linear regression analysis (take obesity as the dependent variable).

Variables	B	S.E	t	p	95% confidence interval
Fasting blood glucose	0.652	0.162	4.025	< 0.001*	0.333–0.970
Hyperlipidemia	1.600	0.465	3.445	0.001*	0.686–2.515
Hypertension	1.173	0.469	2.499	0.013	0.249–2.097

*Means p < 0.05.

In order to verify whether the above conclusions still hold in the normal elderly, we recruited a group of normal controls, and their general demographic data are listed in Table 3. The prevalence of obesity in the normal elderly was 10.3% (16/156), and there was no significant difference (p > 0.05) in age, education, fasting blood glucose, triglyceride, total cholesterol, high-density lipoprotein, low-density lipoprotein, APOE E4, gender, smoker, drinker, tea drinker, physical exercise, hobby, scores of MoCA and GDS between the obesity group and the non-obesity group, what’ more, APOE E4 was not associated with fasting blood glucose, triglyceride, total cholesterol, high-density lipoprotein, low-density lipoprotein, diabetes, hypertension and hyperlipidemia (p > 0.05).

**Table 3:** General demographic data of the Chinese elderly with normal cognitive function based on obesity.

Variables	Obesity (N = 16)	Non-obesity (N = 140)	t or X^2^	p
Age, y	70.63 ± 7.982	69.75 ± 7.589	0.435	0.664
Education, y	8.75 ± 4.669	9.89 ± 3.929	− 1.074	0.285
Fasting blood glucose, mmol/L	5.51 ± 1.099	5.54 ± 1.493	0.180	0.857
Triglyceride, mmol/L	2.88 ± 2.968	1.82 ± 1.136	1.418	0.176
Total cholesterol, mmol/L	5.36 ± 0.979	4.89 ± 1.080	1.652	0.101
High density lipoprotein, mmol/L	1.17 ± 0.332	1.19 ± 0.264	− 0.263	0.793
Low density lipoprotein, mmol/L	3.05 ± 0.902	2.92 ± 0.834	0.611	0.542
BMI, kg/m^2^	30.85 ± 2.455	23.38 ± 2.496	11.356	< 0.001*
Male, n (%)	4 (25.0)	57 (40.7)	1.489	0.285
APOE E4, n (%)	4 (25.0)	21 (15.0)	1.067	0.291
Hypertension, n (%)	8 (50.0)	71 (50.7)	0.003	1.000
Diabetes, n (%)	1 (6.2)	14 (10.0)	0.232	1.000
Hyperlipidemia, n (%)	3 (18.8)	23 (16.4)	0.056	0.732
Smoker, n (%)	2 (12.5)	33 (23.6)	1.011	0.527
Drinker, n (%)	2 (12.5)	28 (20.0)	0.520	0.739
Tea drinker, n (%)	9 (56.2)	59 (42.1)	1.162	0.300
Physical exercise, n (%)	10 (62.5)	91 (65.0)	0.039	1.000
Hobby, n (%)	7 (43.8)	87 (62.1)	2.208	0.182
MoCA	24.13 ± 3.481	25.22 ± 3.759	− 1.113	0.267
GDS	5.69 ± 3.361	5.38 ± 4.444	0.269	0.788

*BMI* body mass index, *MoCA* Montreal Cognitive Assessment, *GDS* Geriatric Depression Scale.

*Means p < 0.05.

## Discussion

To my knowledge, this was the first study to explore the association between APOE E4 and obesity in elderly schizophrenic patients, and we found that (1) The prevalence of obesity in elderly patients with schizophrenia was 15.9%, and its risk factors included elevated fasting blood glucose, hypertension, and hyperlipidemia; (2) APOE E4 was associated with hyperlipidemia, but not was with hypertension or diabetes in elderly schizophrenic patients; (3) APOE E4 was not associated with hyperlipidemia, hypertension or diabetes in the normal elderly.

In our study, we found that the prevalence of obesity in elderly patients with schizophrenia was 15.9%, but there was no difference in the prevalence of obesity between elderly schizophrenics and normal controls. In Yang Tian et al.’s study, they found that the prevalence of obesity in Chinese patients with chronic schizophrenia was 16.4%^25^. In Juan Wang et al.’ study, they found that the prevalence of obesity in patients with schizophrenia was 16.3%, which was not different from that (11.0%) of normal controls^26^. So our conclusions were consistent. However, In Aniyizhai Annamalai et al.’s study^27^, they found that the prevalence of obesity in patients with schizophrenia was 58.5%, which was significantly higher than that (27%) in the population control, and in Mythily Subramaniam et al.’s study, they found that the prevalence of obesity (73.6%) in patients with schizophrenia was significantly higher than that in controls^28^. We speculated that the above differences were mainly due to ethnic differences and different definitions of obesity.

Metabolic syndrome (MetS) is defined by the presence of three or more of the following five criteria: hypertension, hypertriglyceridemia, low HDL cholesterol level, increased waist circumference, and high fasting glucose concentration^29^. A high prevalence of MetS has been reported repeatedly in patients with schizophrenia^30–32^, and it has obvious interaction with obesity^33^. According to the definition of the International Diabetes Federation, abdominal obesity is central to the MetS^34^, while other studies showed that obesity is also a risk factor for metabolic syndrome^35,36^. In the current study, we found that elevated fasting blood glucose, hypertension, and hyperlipidemia were risk factors for obesity in elderly schizophrenics. So our conclusions were consistent, and schizophrenia patients prone to metabolic syndrome and obesity were mainly due to the administration of second-generation antipsychotics^37,38^ and bad living habits, such as smoking, drinking, and lack of exercise^39^.

Next, we explored the association between APOE E4 and obesity in elderly schizophrenic patients. Although we did not find an increased expression of APOE E4 in obese schizophrenic patients, we found that APOE E4 was closely related to hyperlipidemia. However, the above conclusion was not applicable in the normal control group. Utermann et al. found that isoform E4 was significantly more frequent in patients with hypercholesterolemia than normal controls^40^. Maria Odete Rodrigues et al.^41^ found that the epsilon4 allele was more frequent in dyslipidemic than normolipidemic subjects. What’ more, Zhang et al. found that the genotype of apoE4 was associated with higher serum total cholesterol, and APOE levels when compared with the genotypes E3 and E2^42^. So our conclusions were consistent.

Finally, we also discussed the relationship between obesity and neuropsychological tests and psychiatric symptoms in schizophrenic patients, but we did not find that obese patients showed poorer cognitive function or more psychotic symptoms, and the same conclusion was also found in normal elderly people. Nur Amirah Abdul Rashid et al.^43^ found that there was no significant direct effect of BMI on cognition, when cognition was regressed on age, BMI, years of education, and diagnosis of schizophrenia. Depp et al.^44^ found that obesity was associated with worse global cognitive ability in bipolar disorder, but not in schizophrenia. Janney et al. also pointed out that there was no association between obesity and PANSS psychiatric symptoms^45^. However, other studies have shown the opposite conclusion that obesity was a risk factor for cognitive impairment or rich psychiatric symptoms in schizophrenia^46–50^. Therefore, the relationship between obesity and cognitive and mental symptoms needs to be further studied.

Sirtuin1 (SIRT1) belongs to a highly conserved family of protein deacetylase, which is involved in a variety of biological processes, such as cell proliferation, energy metabolism, as well as survival, chromatin dynamics and DNA repair^51^. Previous studies have shown that the *SIRT1* gene may play an important role in the pathophysiology of schizophrenia, bipolar disorder, and Alzheimer's disease, but the exact mechanisms are unclear^52–54^. In recent years, researchers have also proved that *SIRT1* gene regulates obesity and lipid metabolism, for example, Thaddeus et al.^55^ pointed out that *SIRT1* gene may affect obesity by regulating fatty acid oxidation in the liver, influencing obesity-induced inflammation in macrophages, sensing nutrient availability in the hypothalamus, as well as modulating the activity of the circadian clock in metabolic tissues. In addition, the interaction between *APOE* and *SIRT1* may also have an impact on individual’s obesity and cognition^56^, for instance, Jesus Campagna et al.^57^ found that the expression of *APOE E4* would decrease the level of *SIRT1* in serum and brain tissue of AD patients as compared to normal controls; Veena et al.^58^ found that *APOE E4* could reduce the expression of *SIRT1* expression and thus played a neuroprotective role; what’s more, María Teresa Flores-Dorantes et al.^59^ also found that *SIRT1* gene could be affected by *APOE* gene, and then regulated obesity. Therefore, we hypothesized that obesity in patients with schizophrenia may also be influenced by *APOE E4* and *SIRT1* genes, but these conclusions need to be verified in future studies.

There are two limitations to our study. First, this is a cross-sectional study, unable to establish the causal relationship between APOE E 4, cognitive function, and mental symptoms. Second, a relatively small sample size reduces the reliability of the study.

## Conclusions

APOE E4 is associated with hyperlipidemia in schizophrenic patients, which may be a risk factor for obesity in schizophrenic patients, however, the above conclusion does not apply to the normal elderly.

## Data Availability

The datasets used and/or analysed during the current study are available from the corresponding author on reasonable request.

## Abbreviations

APOE: Apolipoprotein E
MoCA: The Montreal Cognitive Assessment
GDS: The Geriatric Depression Scale
PANSS: The Positive and Negative Syndrome Scale
PCR: Polymerase chain reaction
MetS: Metabolic syndrome
BMI: Body mass index
HDL: High density lipoprotein
FDG: Fasting plasma glucose
LDL: Low-density lipoprotein
MCI: Mild cognitive impairment
AD: Alzheimer’s disease
